# Working Alliance in Blended Versus Face-to-Face Cognitive Behavioral Treatment for Patients with Depression in Specialized Mental Health Care

**DOI:** 10.3390/jcm9020347

**Published:** 2020-01-27

**Authors:** Lisa Kooistra, Jeroen Ruwaard, Jenneke Wiersma, Patricia van Oppen, Heleen Riper

**Affiliations:** 1 Department of Clinical, Neuro-and Developmental Psychology and the Amsterdam Public Health Research Institute, Vrije Universiteit Amsterdam, van der Boechorststraat 7, 1081 BT Amsterdam, The Netherlands; 2 Department of Research and Innovation, GGZ inGeest Specialized Mental Health Care, Oldenaller 1, 1081 HJ Amsterdam, The Netherlands; jennekewiersma@gmail.com (J.W.); p.vanoppen@ggzingeest.nl (P.v.O.); 3 Department of Psychiatry and the Amsterdam Public Health Research Institute, GGZ inGeest/Amsterdam UMC, Vrije Universiteit, de Boelelaan 1117, 1081 HV Amsterdam, The Netherlands

**Keywords:** major depressive disorder, blended cognitive behavioral treatment, specialized mental health care, working alliance, randomized controlled trial

## Abstract

This study investigates working alliance in blended cognitive behavioral therapy (bCBT) for depressed adults in specialized mental health care. Patients were randomly allocated to bCBT (*n* = 47) or face-to-face CBT (*n* = 45). After 10 weeks of treatment, both patients and therapists in the two groups rated the therapeutic alliance on the Working Alliance Inventory Short-Form Revised (WAI-SR; Task, Bond, Goal, and composite scores). No between-group differences were found in relation to either patient or therapist alliance ratings, which were high in both groups. In the full sample, a moderate positive association was found between patient and therapist ratings on Task (ρ = 0.41, 95% CI 0.20; 0.59), but no significant associations emerged on other components or composite scores. At 30 weeks, within-and between-group associations between alliance and changes in depression severity (QIDS, Quick Inventory of Depressive Symptomatology) were analyzed with linear mixed models. The analyses revealed an association between depression over time, patient-rated alliance, and group (*p* < 0.001). In face-to-face CBT, but not in bCBT, lower depression scores were associated with higher alliance ratings. The online component in bCBT may have led patients to evaluate the working alliance differently from patients receiving face-to-face CBT only.

## 1. Introduction

Although major depressive disorder (MDD) is a common and severely disabling illness [1], many individuals with MDD have limited access to mental health care and trained therapists [2,3,4]. This has prompted development of interventions requiring less therapist involvement, such as self-help interventions [5,6], and online psychotherapy [7,8]. Various studies have shown that online interventions, most often based on cognitive behavioral therapy, are effective in treating common mental disorders [9,10,11,12,13]—especially when patients receive professional guidance, for example, via secured e-mail exchange or an online treatment platform [5,9,14,15]. The past few years have seen growing interest in hybrid treatment approaches, which either combine or integrate face-to-face and online therapy [16]. As this is a relatively new form of treatment, evidence on effectiveness is still scarce. Initial evaluations suggest that blended treatment can be effective in decreasing depression severity [17,18,19,20,21,22,23,24].

The consequent modifications in the amounts and forms of contact between therapists and patients in online and blended interventions, as compared with face-to-face treatments, have led some therapists to worry that therapeutic alliance might be negatively impacted [18] and that this could weaken treatment outcomes. This is relevant, as therapeutic alliance is considered an important common factor in psychotherapy [25]. A stronger alliance between therapists and patients has been shown to be moderately associated with better treatment outcomes in face-to-face treatment [26,27,28], with meta-analyses reporting overall weighted averages of *r* = 0.28 (95% CI 0.26; 0.30) in psychotherapy for mental disorders [28] and *r* = 0.26 (95% CI 0.19; 0.32) in cognitive behavioral therapy (CBT) for depression [29]. However, such associations might be moderated by other factors. For example, assessment of alliance in a later phase of treatment may detect stronger associations between alliance and treatment effect than early assessment [28,29], and the alliance–outcome association has been found weaker for therapist-rated alliance than patient-rated alliance [29]. Associations might also be positively impacted if patient and therapist ratings of working alliance more strongly concur [30], or if patients have had fewer prior depressive episodes [31].

For blended depression treatments, available evidence on therapeutic alliance is still limited. Initial evaluations showed patient-rated alliance in online interventions to be high and comparable to ratings in face-to-face treatment [32,33]. While the therapeutic alliance in guided online interventions is often assumed to be less salient to outcome [9], Flückiger and colleagues [28] have suggested that the overall alliance–outcome association in online therapy is comparable to the association in face-to-face treatment (*r* = 0.28, 95% CI 0.21, 0.34, *p* < 0.001), with higher alliance accompanying better treatment outcomes. A recent review has suggested that the association between alliance and outcome in online interventions may be stronger in terms of agreement on goals and tasks than in terms of the affective bond between patients and their therapists, partly because patients could have lower or different expectations of the bond that can be formed online [32]. In a recent Swedish study (*n* = 73), patients and therapists provided high alliance ratings for blended CBT [34]. The working alliance as rated by therapists, but not as rated by patients, was found predictive of changes in depression severity during treatment. Among the possible explanations for this finding, the authors suggest that therapists in blended cognitive behavioral therapy (bCBT) might have provided more accurate evaluations of working alliance than patients, because patients would have based their alliance ratings on both the online (self-help) material and the face-to-face contacts, whereas therapists would have rated the interaction with the patient in face-to-face contacts only.

The present study expands on existing knowledge by examining working alliance in a randomized controlled trial (RCT), with comparison of blended cognitive behavioral therapy (bCBT) to face-to-face CBT in patients with major depressive disorder (MDD) requiring specialist mental health care. Information on the working alliance was gathered from both patients and therapists, in order to gain insight into the dyadic nature of this relationship [30]. Data was collected in an RCT focusing on the cost-effectiveness of bCBT versus face-to-face CBT [24,35]. The blended intervention integrated face-to-face and online CBT into a single treatment protocol [20,24,35], replacing half of the face-to-face sessions by online sessions. This paper addresses three research questions: (1) Is there a difference between bCBT and face-to-face CBT in terms of patient and therapist ratings of working alliance? (2) Is there an association between working alliance and change in depressive symptoms? and (3) If so, does that association differ between bCBT and face-to-face CBT?

## 2. Materials and Methods

### 2.1. Study Design

Data were collected between August 2014 and May 2017 within a pilot randomized controlled trial (*n* = 102), that compared bCBT with face-to-face CBT in six outpatient specialized mental healthcare clinics. The total RCT sample consisted of 102 participants aged 19–62. A detailed overview of the design and procedures of the trial can be found elsewhere [35]. The trial was registered at the Netherlands Trial Register (number NTR4650) and was approved by the Medical Ethics Committee of the Vrije Universiteit Medical Center (VUmc) (registration number 2014.191).

### 2.2. Participants

Patients for the trial were recruited during the intake procedure before the start of their specialized depression treatment. All patients had a primary diagnosis of MDD, based on the Mini-international Neuropsychiatric Interview Plus (MINI-plus, [36,37]), and had adequate proficiency in the Dutch language, a valid e-mail address, and a computer with Internet access. Patients with a high risk for suicide, a psychotic disorder, a bipolar disorder, or substance dependence were excluded from study participation. No exclusion criteria were applied regarding treatment histories, other comorbidity, or parallel pharmacological treatments. The current study included patients for whom patient-rated or therapist-rated alliance at week 10 was available (*n* = 92; bCBT *n* = 45, CBT *n* = 47).

### 2.3. Procedures

Patients were asked to provide written informed consent in order to participate in the study. Demographic data, treatment preference, and diagnostic profiles were assessed prior to random allocation and start of treatment (baseline). Working alliance was assessed ten weeks after the start of treatment. At this time point, both groups ideally would have received ten face-to-face sessions. The bCBT group was expected to have received nine additional online sessions and eight online therapist feedback messages. Patients completed weekly assessments on their depression severity during their treatment. Assessments at baseline and after ten weeks of treatment consisted of online self-report questionnaires and a diagnostic interview, administered by trained assessors who were blinded to treatment allocation.

### 2.4. Random Allocation

Random allocation (1:1 ratio) was performed by an independent researcher, based on a computer-generated random number table. Random allocation was stratified per treatment center. Patients began treatment approximately three weeks after random allocation (SD 2.0, range 0 to 14).

### 2.5. Interventions

Depression treatment in both study arms was based on CBT manuals [38,39], which advise therapists to provide 15 to 20 weekly CBT sessions and to include psycho-education, behavioral activation, cognitive restructuring, and relapse prevention. The face-to-face CBT without the online sessions was provided at the outpatient clinics. Therapists were advised to plan weekly sessions but were allowed to deviate from the treatment manual when necessary. Duration of treatment could vary per patient, but was expected to last approximately 20 weeks.

Blended CBT consisted of ten face-to-face sessions at the clinic and nine online sessions. Face-to-face and online sessions were alternated. Therapists had received training in the use of the bCBT manual. The aim was to schedule one face-to-face, one online session, and one online feedback message per week during a ten-week period, beginning with a face-to-face session. Patients planned the sessions together with their therapist. Each session focused on a specific domain of the CBT protocol, such as psycho-education, activity scheduling, and identification of dysfunctional assumptions. The content of the online sessions corresponded with that of the previous face-to-face session. Sessions were provided in a fixed order, but therapists were allowed to repeat an online session. In order to access the web-based part of treatment, patients and therapists logged into a personal account on a secure website (www.minddistrict.com). The online sessions consisted of text, short videos, images, patient vignettes, and homework exercises. Patients also had access to a daily mood diary, weekly monitoring of depression severity (QIDS-SR, Quick Inventory of Depressive Symptomatology, Self-Report version) [40], and a messaging function to contact their therapists. Therapists provided a written therapeutic feedback message to each completed online session. Feedback was asynchronous and was provided within three working days after patients had completed the online session. After reading their therapist’s feedback, patients gained access to the next online session. More detailed information on the form and content of bCBT can be found elsewhere [20,35].

### 2.6. Therapists

A total of 33 therapists participated in the trial, 5 of whom only provided face-to-face CBT, 12 only provided bCBT only, and the remaining 16 therapists treated patients in both treatment arms. On average, therapists treated three patients (SD 2.5, range 1 to 10). All therapists had at least a master’s degree in psychology, were specialized in cognitive behavioral treatment, and had a minimum of two years of relevant work experience.

### 2.7. Measures

In assessing therapeutic alliance, this study focused on Bordin’s definition of working alliance [41], which refers to the *bond* between patient and therapist as well as agreement about the *goals* and *tasks* in treatment. Patients and therapists were asked separately to assess their working alliance with the abbreviated Dutch version of the Working Alliance Inventory Short-form Revised (WAI-SR) questionnaire [42,43,44]. Both questionnaire versions were scored on a five-point Likert scale, ranging from “never” to “always”. Items are grouped to measure the three components of working alliance: agreement on Goals, agreement on Tasks, and the quality of the Bond between therapist and patient [41]; a composite (average) score is also calculated as a global assessment of working alliance. Examples of items are “We are working towards mutually agreed upon goals” for the Goal component; “I believe the way we are working with my problem is correct” for the Task component; and “We respect each other” for the Bond component. The client version (WAI-SR-C) consists of 12 items [45], the therapist version (WAI-SR-T) of 10 items. For descriptive purposes, patient and therapist ratings were transformed to a 0 to 5 range for each component. In the current study, both versions of the WAI displayed good internal consistency. Cronbach’s alpha for the patient version was 0.91 (95% CI 0.89; 0.94) for the composite score (all items), 0.86 (95% CI 0.82; 0.91) for the Goal component, 0.90 (95% CI 0.86; 0.93) for the Task component, and 0.82 (95% CI 0.77; 0.87) for the Bond component. For the therapist version, Cronbach’s alpha was 0.88 (95% CI 0.84; 0.91) for the composite score, 0.81 (95% CI 0.74; 0.87) for the Goal component, 0.81 (95% CI 0.74; 0.87) for the Task component, and 0.79 (95% CI 0.71; 0.85) for the Bond component.

Change in self-reported depression severity was assessed with the Dutch translation of the 16-item short version of the Inventory of Depressive Symptomatology (QIDS-SR) [40]. Patients were requested to complete the QIDS on a weekly basis during treatment. The questionnaire covers all nine components of the DSM-IV-TR (Diagnostic and Statistical Manual of Mental Disorders, fourth edition, text-revision) depression diagnosis. Total scores range between 1 and 27. In the current study, the Cronbach’s alpha at baseline was 0.78 (95% CI 0.71; 0.84).

Before random allocation, demographic information, including age, gender, employment status, and income, was collected with an online self-report questionnaire at baseline. One further item was added to inquire whether patients would prefer receiving bCBT or face-to-face CBT. Diagnosis of depression and psychiatric comorbidity were obtained using the Mini-international Neuropsychiatric Interview Plus (MINI-plus, [36,37]). Information on face-to-face treatment uptake (number of sessions completed) was retrieved from patient records. Information on the number of feedback messages received and online sessions completed by patients was obtained from logfiles from the online treatment environment.

### 2.8. Statistical Analyses

The current study was based on a data subset from a larger trial (*n* = 102) [24,35], and was therefore not powered a priori. The study sample (*N* = 92) was large enough to detect a correlation of r ~ 0.3 and a group difference of d ~ 0.6 at a power of 80% and a significance level of *p* = 0.05.

Multivariable logistic regression analysis including age, gender, partner status, baseline depression severity, comorbidity, treatment group, a priori treatment preference, and total number of face-to-face sessions received was performed to examine possible differences between patients that did and did not provide working alliance ratings.

Between-group differences in patient and therapist ratings of the separate work alliance components were examined using Welch Two-Sample t-test (*p* < 0.05), with patient and therapist ratings as the dependent variable and treatment group as the independent variable.

The degree of consensus between patients and therapists was examined by calculating correlations between patient and therapist ratings. Shapiro–Wilk tests, at alpha = 0.05 [46], revealed non-normal distributions for all separate components, except for the patient ratings on the Task component. We therefore assessed Spearman’s rho (for all component pairs. Correlations were estimated using the psych package (version 1.8.4) [47] in R software (Macintosh; Intel Mac OS X v3.5.1) [48]. The relationship between change in depression severity over time, treatment group, and patient- and therapist-rated working alliance was assessed in separate linear mixed-effect models (LMM) with restricted maximum likelihood (REML). The LMM approach was chosen in order to account for missing QIDS data and the correlation between follow-up time points. Because the Goal, Task, and Bond components were moderately to highly intercorrelated (range Task~Bond = 0.41 to Task~Goal = 0.70), only the composite scores were used to examine the association between general working alliance and outcome. The models thus employed QIDS depression severity scores as the independent variable and time (in weeks), treatment group, and the WAI composite scores as dependent variables. Composite scores were centered. The models also included a random effect for individuals over time, which allowed for estimation of individual intercepts and slopes [49,50]. The Holm–Bonferroni correction [51] was applied to account for multiple testing. In the manuscript, noncorrected nonsignificant *p*-values are reported for descriptive purposes. The mixed models were estimated using the lmer function from the lme4 package (version 1.1–17) [52] in R software (Macintosh; Intel Mac OS X, v3.5.1) [48].

## 3. Results

### 3.1. Patients

From the full trial sample of 102 patients (bCBT *n* = 53, CBT *n* = 49), data on working alliance was available for 92 patients (bCBT *n* = 45, CBT *n* = 47). Both the patient and therapist ratings had been obtained for 71 of them (bCBT *n* = 38, CBT *n* = 33), whereas solely therapist ratings were obtained for 19 patients (bCBT *n* = 8, CBT *n* = 11) and patient ratings for 2 patients (bCBT *n* = 1, CBT *n* = 1). Information on baseline demographic and clinical characteristics of patients included in this study *(N* = 92) is presented in Table 1.

### 3.2. Study Dropout

Gender, age, relationship status, employment, treatment preference, comorbidity, depression severity, and treatment group were not significantly related to missing working alliance ratings (*p* > 0.5). Patients who received fewer face-to-face sessions during the first ten weeks of treatment had higher odds of not providing information on working alliance (β = −0.849, OR = 0.43, 95% CI: 0.23; 0.64, *p* < 0.001). In addition, there was a high overlap between study dropout and treatment dropout; six out of nine patients who did not provide data also did not receive any face-to-face sessions.

### 3.3. Treatment Received

Table 2 provides an overview of treatment received before and after the alliance ratings (ten weeks after start of treatment). Before rating therapeutic alliance, patients in the bCBT group had received an average of 7.1 (SD 2.1) face-to-face sessions and 7.9 (SD 2.4) online sessions; those in the face-to-face CBT group had received a mean of 6.6 (SD 2.2) sessions by week 10. Average per-patient therapist time, including time spent on online feedback, during the first ten weeks of treatment was 636 min (SD 187) in bCBT and 395 min (SD 132) in face-to-face CBT. This difference was statistically significant—95% CI −308.7; −173.5, *t* (90) = −7.08, Holm-corrected *p* < 0.001. Over the full study period, therapist time between the two groups did not significantly differ (bCBT 944 min SD 274 versus CBT 844 min SD 348, *p* = 0.130). Overall, the face-to-face CBT group received more sessions at the clinic than the bCBT group—14.1 sessions (SD 5.8) versus 11.1 sessions (SD 3.5); mean difference 3.0, 95% CI 1.0; 5.0, *t* (71.23) = 2.99, *p* = 0.004, Holm-corrected *p* = 0.008).

Comparing the total amount of treatment received in both groups with the planned amount of sessions based on the bCBT and CBT treatment protocols, we found that the bCBT group received on average 111% of the planned sessions (averaging one additional face-to-face session and one additional online session) and the CBT group received 70% to 94% (14 out of 15 to 20 planned sessions).

### 3.4. Working Alliance

After applying a Holm–Bonferroni correction [51] to account for multiple testing, we found no statistically significant differences between treatment groups on either the patient-rated or the therapist-rated composite scores, and also not on the separate Task, Goal, and Bond components of working alliance. Results are shown in Table 3.

Linear regression analyses showed no significant differences between treatment groups in the associations between patient and therapist evaluations of Task and Goal. In terms of the Bond and the composite scores, positive therapist evaluations appeared to have stronger associations with more positive patient evaluations in the bCBT group, but these were not significant after correction for multiple testing (Bond: 95% CI 0.16; 1.72, *p* = 0.019, Holm-corrected *p* = 0.077; composite: 95% CI −0.0; 1.49; *p* = 0.053, Holm-corrected: *p* = 0.160).

Controlling for therapist time invested in treatment in explorative linear regression analyses led to similar outcomes as described above in the uncontrolled analyses, showing nonsignificant between-group differences in patient-rated alliance, therapist-rated alliance, and associations between patient and therapist evaluations (*p* > 0.05).

In the full sample, patient and therapist composite scores were not significantly related, nor were ratings with regard to the Goal and Bond components. On the Task component, a significant moderate correlation of ρ = 0.41 was found (95% CI: 0.20; 0.59, *p* < 0.001, Holm-corrected *p* = 0.001). In order to gain further insight into the level of agreement between patients and therapists, a difference score was calculated by subtracting the (recoded) therapist composite score from the patient composite score (range 0 to 5). Results showed that 43 out of 71 patients (61%) gave lower ratings than their therapists (mean −1.1, range −2.2 to −0.3); 14 patients (20%) gave similar ratings (mean −0.1, range −0.2 to 0.2), and 11 patients (16%) gave more positive evaluations (mean 0.7, range 0.3 to 4.0).

Explorative linear regression models assessing the relationship between therapist time and the therapist and patient ratings revealed only a significant positive association between therapist time and therapists’ ratings of the Task component. This relationship remained significant after controlling for treatment condition (*t* (85) = 2.84, *p* = 0.006, Holm-corrected *p* = 0.023).

### 3.5. Working Alliance and Depression Severity

**Figure 1:**
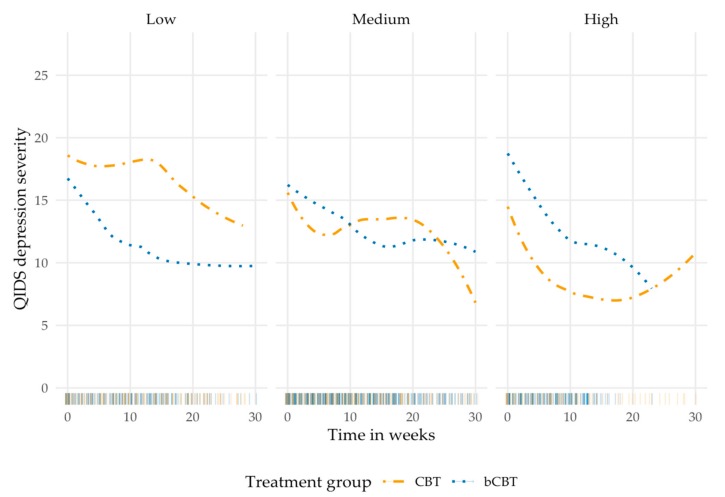
Observed change in depression over time for both treatment groups, clustered in low, medium, and high patient-rated working alliance (measured at week 10). bCBT: blended Cognitive Behavioral Therapy; CBT: Cognitive Behavioral Therapy (face-to-face only); QIDS: Quick Inventory of Depressive Symptomatology.

On average, patients completed 22.7 (SD 8.6, range 1 to 42) QIDS measurements. In the week before the assessment of working alliance at ten weeks, the mean QIDS depression severity score was 13.3 (SD 6.2, range 1 to 27), indicating moderately severe depression. Visual inspection of change in depression severity over time suggested a curvilinear pattern. Therefore, linear and quadratic trends were tested in a polynomial model. Adding the polynomial terms to the model led to a better model fit (*p* < 0.001). For patient-rated working alliance (composite score), there was a significant three-way interaction between both the linear and quadratic trends, treatment group and alliance—linear: *b* = 0.61 (95% CI 0.34; 0.88), *t* (169.50) = 4.25, Holm-corrected *p* < 0.001, quadratic: *b* = −0.03 (95% CI −0.04; −0.02), *t* (1028) = −5.34, Holm-corrected *p* < 0.001. In Figure 1, the association is visualized by rounding centered alliance scores to low (−1, *n* = 21, range 2.0 to 3.0), medium (0, *n* = 32, range 3.1 to 3.9), and high alliance (1, *n* = 21, range 4.0 to 5.0). Combined with weekly QIDS data, there were 320, 543, and 255 data points available in the respective categories. Density of data points is displayed along the *x*-axes of the three graphs. The direction of the regression lines after 20 weeks should be interpreted cautiously, because fewer data points were available after that time point.

The graphs in Figure 1 suggest that depression severity in the face-to-face CBT group was associated with patient evaluations of working alliance. In the bCBT group, a similar pattern of decreasing depression severity appeared to occur in all three alliance categories. Post hoc subgroup analyses supported this theory. In bCBT, no alliance–outcome association was found. In the face-to-face CBT group, patients who provided higher ratings had significantly lower QIDS scores—*b* = −7.78 (95% CI −13.10; −1.95), *t* (27.21) = −2.68, uncorrected *p* = 0.012—than patients giving lower ratings. Control for therapist time invested in treatment did not alter the findings. Table 4 shows the results of the linear mixed model assessing patient-rated working alliance.

Therapist-rated alliance was not significantly associated with outcome (*p* > 0.05). LMM revealed similar significant interactions between treatment group and the linear and quadratic trends, suggesting a steeper decrease in depression severity during the first ten weeks of treatment in the bCBT group versus a more linear pattern of decrease of severity in the face-to-face CBT group. Rounding centered alliance scores for descriptive purposes showed that therapists provided lower overall alliance ratings in 2 cases (range 2.9 to 3.0), medium ratings in 58 cases (range 3.1 to 3.9), and high ratings in 30 cases (range 4.0 to 5.0). Explorative analysis revealed no association between the level of agreement (convergence) between patients’ and therapists’ working alliance ratings and outcome (*p* > 0.05).

## 4. Discussion

This study examined working alliance between patients and their therapists in blended cognitive behavioral therapy (bCBT) for depression, as provided in specialized mental healthcare settings, and compared it with the working alliance in face-to-face CBT.

### 4.1. Working Alliance between Patients and Therapists

No differences were found between treatment groups in the way patients and therapists evaluated the working alliance, nor in the associations between patient and therapist ratings of working alliance within either group. This suggests that providing part of treatment online, rather than face-to-face, did not have a negative impact on working alliance between patients and therapists. This is an important finding, because prior research found that therapists voiced this as one of their concerns when considering blended treatment [18]. In general, patients and therapists in both treatment groups were satisfied with the working alliance. Because the current study was one of the first to examine working alliance in bCBT compared with face-to-face CBT, these results cannot be related to outcomes from other studies yet. The high alliance ratings are similar to those reported in other studies that examined patient- and therapist-rated alliance, for example, the trial by Preschl and colleagues (*N* = 53) which compared online CBT with face-to-face CBT for depression [33], and the uncontrolled study by Vernmark and colleagues (*N* = 73) in blended CBT for depression [34].

While both patients and therapists provided positive ratings on average in the current study, no significant association was found between patient and therapist evaluations on general alliance (composite score), and on the Goal and Bond components. On the Task component a moderate positive association was found in the full study sample (ρ = 0.41, 95% CI: 0.20; 0.59), suggesting that higher patient ratings on the Task domain concurred with higher therapist ratings. Further exploration of general working alliance showed that over half of the patients (43 out of 71, 61%) provided less positive evaluations than their therapist, with an average of minus 1 point on a five-point scale. While this is a notable finding, the level of agreement between patients and therapists was not associated with change in depression severity or vice versa. This suggests the degree of convergence after ten weeks of treatment did not impact the treatment effect in the current study.

### 4.2. Association between Working Alliance and Depressive Symptoms

For patient-rated alliance, an alliance–outcome association was found in the face-to-face group, but not in the bCBT group. In face-to-face CBT, higher depression severity was accompanied by lower alliance ratings, and vice versa. The results for bCBT concurred with the findings of the above-cited study by Vernmark and colleagues [34], which likewise found no alliance–outcome association for patient-rated working alliance in bCBT. That study, however, did not include a comparison group in the analyses. The cause of the disparity in the alliance–outcome association between our study groups cannot be determined here, and more research is needed. Because patients in both groups received CBT, the difference in the alliance–outcome association is more likely to be related to treatment *form* rather than to *content* or therapeutic techniques. One possible explanation is that in bCBT, more emphasis was placed on self-efficacy and autonomy by letting patients work through part of the treatment protocol on their own via the online platform. Hence, patients in bCBT were possibly less dependent on their therapist to achieve a change in depression severity than patients in the face-to-face CBT group.

In this study, no association between therapist-rated alliance and outcome was found in both groups. A possible explanation is that therapists were predominantly positive about the working alliance with their patients, thus restricting variance in this variable and limiting the feasibility of detecting associations between change in depression severity and working alliance. Comparison to other work is complicated, as therapist ratings are often not included in studies. The meta-analytic synthesis by Flückiger and colleagues [28], for example, identified 295 study samples that examined alliance in adult psychotherapy for various mental health problems, but only 40 (14%) of these included therapist ratings. Overall, the synthesis found similar alliance–outcome associations for the alliance ratings of both patients and therapists, suggesting that our current findings are not consistent with general findings on this issue. The same holds for the findings in the Vernmark study, where a significant association was found for therapist ratings and outcome in bCBT, but not for patient ratings, with each point increase in therapist ratings being associated with a 0.5 point reduction per week on the PHQ-9 (Patient Health Questionnaire-9) (95% CI −0.74; −0.26).

### 4.3. Change in Depressive Symptoms

Over the full study period (30 weeks), both of our treatment groups showed a similar overall improvement in depressive symptoms. Compared with patients in standard face-to-face CBT, those in bCBT reported a steeper decrease in depression severity in the first fifteen weeks of treatment, after which depression scores stabilized. In the face-to-face group, a more linear pattern was observed. This difference is an indication that the higher treatment intensity in bCBT might lead to additional health benefits. Our planning of bCBT treatment intensity was based on the 2013 meta-regression analysis by Cuijpers and colleagues [53], which focused on the amount of psychotherapy required to treat depression. The study suggested that, rather than treatment duration and dosage, the intensity in which sessions are offered per week positively impacts the effect of treatment. While the original goal of providing 18 bCBT sessions in ten weeks [35] was not met in the current study, patients did receive an average of 78% of treatment during that time frame, while those in the less intensive face-to-face CBT group received 37% of the treatment protocol during this period. However, such results should be interpreted with caution, as the current study was not designed to specifically examine the relationship between treatment intensity and treatment effects. Future studies should explore this further, along with the possible role a blended approach could play in achieving higher effectiveness through higher intensity.

### 4.4. Strengths and Limitations

The current study was one of the first to examine working alliance in a blended cognitive behavioral treatment format in routine practice, and to compare it with alliance in standard face-to-face CBT. By assessing depression severity at a weekly basis, the study also sheds light on the changing severity of depression over the course of treatment, revealing patterns of change rather than absolute differences measured before and after therapy. Moreover, the study included both patient and therapist evaluations of working alliance, providing insights into the degree of convergence between the actors and its possible effects on treatment outcome.

There are also some limitations to be considered while interpreting the results. First, working alliance was treated as a stable factor and was therefore measured only once. That time point was chosen in relation to the expected ten-week duration of the bCBT protocol. Future studies could consider performing an additional measurement at an early stage of treatment, or integrating a weekly assessment of working alliance into the treatment. That would help clarify changes and dynamics in therapeutic alliance over time. Repeated measures would enable more detailed comparison of patterns of depression severity and alliance ratings.

Second, the study had a relatively small sample size, which limited the power to detect small to moderate effects. These have therefore not been revealed by our study. Larger studies could also examine factors that potentially moderate or mediate associations, such as patient demographics, number of prior episodes, and prior experiences with treatment.

Third, the current study specifically focused on patients in outpatient special mental health care for depression. In order to further establish the potential value of bCBT, it is important to evaluate bCBT in other settings, such as primary care, and in different countries. Results from a large European study are forthcoming, examining the comparative effectiveness of bCBT versus treatment as usual in eight different European countries [16].

Additionally, it would be interesting to differentiate between online and face-to-face working alliance within bCBT. Patients might have different expectations of agreement on goals and tasks in the face-to-face sessions than in the online sessions. In the current study, for example, patients and therapists could modify general tasks and goals during their face-to-face sessions, while the content of the online sessions was fixed. Further, it would be interesting to examine whether patients evaluate the online affective bond differently from the bond with their therapist.

Fourth, 16 out of 33 therapists in our study treated patients in both treatment arms, allowing them to compare their experiences with both types of treatment. This could potentially cause bias. Because of the limited sample size, no subgroup analyses were done to examine a possible effect on the evaluation of working alliance when therapists treat patients in both treatment groups.

Finally, because this was one of the first studies to examine bCBT, therapists were relatively inexperienced with the format. It would be interesting to examine whether working alliance ratings in blended treatment increase with the therapists’ level of experience.

## 5. Conclusions

This study shows that bCBT and face-to-face CBT are associated with similarly high working alliance ratings by both patients and therapists when provided to patients with depression in specialized mental health care. Replacing a proportion of the face-to-face sessions with online sessions and online therapist feedback evidently has no negative effect on working alliance and treatment effect. We did find that a more positive evaluation of working alliance was associated with lower depression severity in face-to-face CBT, whereas no such alliance–outcome association was seen in bCBT. The reason for that difference between treatment groups is still unclear. Replication of these results in larger samples to also enable assessment of possible moderators and mediators is warranted.

## Figures and Tables

**Table 1:** Sample characteristics at baseline.

	bCBT *n* = 47	CBT *n* = 45	Total *n* = 92
Demographic			
Gender, female (*n*, %)	30 (63.8)	25 (55.6)	43 (59.8)
Age (mean, SD)	39.5 (11.4)	37.7 (10.5)	38.6 (11.0)
In a relationship (*n*, %)	26 (55.3)	27 (60.0)	53 (57.6)
Education (*n*, %)			
Low	6 (12.8)	1 (2.2)	7 (7.6)
Middle	24 (51.1)	31 (68.9)	55 (59.8)
High	17 (36.2)	13 (28.9)	30 (32.6)
Employed (*n*, %)	27 (57.5)	26 (57.8)	53 (57.6)
Nationality, Dutch (n, %)	42 (89.4)	44 (97.8)	68 (93.5)
			
Clinical			
Treatment preference, bCBT (*n*, %)	30 (63.8)	30 (66.7)	60 (65.2)
Co-morbid disorder(s) (*n*, %)	31 (66.0)	30 (66.7)	61 (66.3)
QIDS-SR (mean, SD)	16.6 (4.9)	15.9 (4.1)	16.3 (4.6)
Antidepressant medication (*n*, %)	32 (68.0)	27 (60.0)	59 (64.1)

Notes: bCBT: blended cognitive behavioral therapy; CBT: cognitive behavioral treatment (face-to-face only); QIDS-SR: Quick Inventory of Depressive Symptomatology, Self-Report version; SD: Standard deviation.

**Table 2:** Treatment received in the two study groups.

	bCBT n = 47	CBT n = 45	Total n = 92
Treatment received before assessment of working alliance: mean (SD)			
Face-to-face sessions	7.1 (2.1)	6.6 (2.2)	6.7 (2.2)
Online sessions	7.9 (2.4)	-	-
Online feedback messages	6.9 (2.5)	-	-
Therapist time in minutes	636 (187)	395 (132)	518 (203)
			
Treatment received after assessment of working alliance: mean (SD)			
Face-to-face sessions	4.0 (2.7)	7.5 (4.8)	5.7 (4.3)
Online sessions	2.7 (2.8)	-	-
Online feedback messages	2.4 (2.6)	-	-
Therapist time in minutes	308 (208)	449 (289)	377 (258)
			
Treatment received during the full study period: mean (SD)			
Face-to-face sessions	11.1 (3.5)	14.1 (5.8)	12.5 (5.0)
Online sessions	10.3 (2.9)	-	-
Online feedback messages	9.3 (3.5)	-	-
Therapist time in minutes	944 (274)	844 (348)	895 (315)

Notes: bCBT: blended cognitive behavioral therapy; CBT: cognitive behavioral treatment (face-to-face only).

**Table 3:** Patient- and therapist-rated working alliance in the two treatment groups.

	Patients				Therapists			
				Mean difference				Mean difference
				(95% CI)				(95% CI)
Outcome ^1^	bCBT	CBT	Total	bCBT vs. CBT	bCBT	CBT	Total	bCBT vs. CBT
	*n* = 39	*n* = 34	*n* = 73		*n* = 46	*n* = 44	*n* = 90	
Goal	16.3 (3.1)	16.1 (3.2)	16.2 (3.1)	0.25 (−1.22; 1.71)	12.4 (1.9)	11.7 (1.8)	12.1 (1.9)	0.73 (0.04; 1.50) ^a^
Scaled	4.1 (0.8)	4.0 (0.8)	4.1 (0.8)		4.1 (0.6)	3.9 (0.6)	4.0 (0.6)	
Task	12.4 (4.1)	11.9 (4.2)	12.2 (4.1)	0.47 (−1.45; 2.40)	12.1 (2.0)	11.2 (1.6)	11.7 (1.9)	0.93 (0.17; 1.68) ^b^
Scaled	3.1 (1.0)	3.0 (1.1)	3.0 (1.0)		4.0 (0.7)	3.7 (0.5)	3.9 (0.6)	
Bond	15.6 (3.3)	14.7 (3.6)	15.2 (3.4)	0.97 (−0.64; 2.57)	17.9 (1.9)	17.6 (2.1)	17.7 (2.0)	0.28 (−0.52; 1.17)
Scaled	3.9 (0.8)	3.7 (0.9)	3.8 (0.9)		4.6 (0.5)	4.4 (0.5)	4.4 (0.5)	
Composite	3.7 (0.7)	3.6 (0.8)	3.6 (0.8)	0.14 (−0.22; 0.50)	4.2 (0.5)	4.0 (0.4)	4.1 (0.5)	0.20 (0.00; 0.40) ^c^

Notes: ^1^ Data are presented as mean (standard deviation). bCBT: blended cognitive behavioral therapy; CBT: cognitive behavioral treatment (face-to-face only). ^a^ uncorrected: *p* = 0.063, Holm-corrected: *p* = 0.144; ^b^ uncorrected: *p* = 0.016, Holm-corrected: *p* = 0.063; **^c^** uncorrected: *p* = 0.048, Holm-corrected: *p* = 0.424.

**Table 4:** Results of the linear mixed model with patient-rated working alliance.

	Dependent Variable				
Predictors	Estimates	SE	95% CI	Statistic	*p*-value
(Intercept)	14.96	0.88	13.25; 16.68	17.08	<0.001
Time × group × working alliance	0.61	0.14	0.33; 0.89	4.25	<0.001
Quadratic time × group × working alliance	−0.03	0.00	−0.04; −0.02	−5.34	<0.001

Notes: SE: Standard error. Random effects: residual (σ^2^) = 6.63; intercept (τ_00_) = 20.70; slope (τ_11_) = 0.08; intercept–slope covariance (ρ_01_) = −0.17; intraclass correlation coefficient (ICC) = 0.76; *N* = 70; Observations 1118; Marginal *R*^2^/Conditional *R*^2^ = 0.191/0.804; log likelihood = −2861.15.
